# etymologia: Cryptococcus neoformans

**DOI:** 10.3201/eid1405.0508

**Published:** 2008-05

[krip′′ to-kok′əs ne′′o for-mənz], from the Greek—*krypto* (hidden), *kokkos* (berry), *neos* (new); and Latin—*forma* (form)

*C. neoformans* is an encapsulated yeastlike fungus of the family *Cryptococcaceae. *It was first described in 1894 by German pathologist Otto Busse, who observed the cells in a tumor from the tibia of a woman with sarcoma. Found worldwide in nests and droppings of pigeons, it is the most common species that causes cryptococcosis in humans. The effects range from asymptomatic infection to meningitis, pneumonia, or disseminated disease. The crucial factor is the immune status of the host. With the global emergence of AIDS, the incidence of cryptococcosis is increasing and now represents a major life-threatening infection in these patients.

